# Prediction Methods of Herbal Compounds in Chinese Medicinal Herbs

**DOI:** 10.3390/molecules23092303

**Published:** 2018-09-10

**Authors:** Ke Han, Lei Zhang, Miao Wang, Rui Zhang, Chunyu Wang, Chengzhi Zhang

**Affiliations:** 1 School of Computer and Information Engineering, Harbin University of Commerce, Harbin 150028, China; 2 Life Sciences and Environmental Sciences Development Center, Harbin University of Commerce, Harbin 150010, China; 13212921382@163.com (L.Z.); w1993817m@163.com (M.W.); yiyangchunxiao@163.com (C.Z.); 3 College of Tourism and Landscape Architecture, Guilin University of Technology, Guilin 541001, China; sophiewu@yahoo.com; 4 School of Computer Science and Technology, Harbin Institute of Technology, Harbin 150001, China; chunyu@hit.edu.cn

**Keywords:** Chinese herbal compounds, drug-likeness, virtual screening, comparative study

## Abstract

Chinese herbal medicine has recently gained worldwide attention. The curative mechanism of Chinese herbal medicine is compared with that of western medicine at the molecular level. The treatment mechanism of most Chinese herbal medicines is still not clear. How do we integrate Chinese herbal medicine compounds with modern medicine? Chinese herbal medicine drug-like prediction method is particularly important. A growing number of Chinese herbal source compounds are now widely used as drug-like compound candidates. An important way for pharmaceutical companies to develop drugs is to discover potentially active compounds from related herbs in Chinese herbs. The methods for predicting the drug-like properties of Chinese herbal compounds include the virtual screening method, pharmacophore model method and machine learning method. In this paper, we focus on the prediction methods for the medicinal properties of Chinese herbal medicines. We analyze the advantages and disadvantages of the above three methods, and then introduce the specific steps of the virtual screening method. Finally, we present the prospect of the joint application of various methods.

## 1. Introduction

The main source of Chinese herbal compounds is plants that are widely found in nature. Afterwards a series of extraction and chemical synthesis occurs, leading to a wider range of Chinese herbal compounds [1,2,3]. Under a broader definition, the source of Chinese herbal compounds is far wider than that. For the traditional Chinese herbal medicine preparation process, we need to use the processing technology. In the book “Processing Guide” by Zhang Rui, a medical scientist in the Qing Dynasty in China, there are records of processing Chinese herbal medicines by baking, cutting, frying, washing, soaking, bleaching, steaming, boiling, etc. [4]. The purpose is to eliminate or reduce the toxicity of the drug, enhance the efficacy, facilitate preparation and storage, and make the drug pure. It has been gradually found through modern Chinese pharmacy research that the processing technology does not only affect the traits of Chinese medicinal materials [5]. Through different processing techniques, a variety of active compounds in Chinese herbal medicines also undergo different chemical reactions, resulting in a wider range of active compounds. Similarly, in the process of decoction of traditional Chinese medicine, many active ingredients in the traditional Chinese medicine compound are dissolved in water, and these ingredients also undergo various chemical reactions in water to produce more compounds [6,7]. As far as it is known, Chinese herbal medicines often contain more than one hundred active compounds. For example, the number of active compounds currently known in the traditional Chinese medicine compound Liuwei Dihuang Pill is 183 [8]. Of course, in such a large number of active compounds of traditional Chinese medicine, the active ingredients and toxic ingredients coexist. How to find a better and faster method to predict the drug-like properties of the active compounds of traditional Chinese medicines? This has become an important task for Chinese medicine R&D workers in the new era.

On the other hand, the current Chinese herbal medicine-derived compounds have been widely accepted as a source of lead compounds in the development of chemical drugs. Studies have confirmed that compounds derived from Chinese herbal medicines are more active than compounds of other sources [9]. SimhadriVsdna N. et al. studied Sole shine formulations for human dermatophytosis, a mixture of various plant extracts [10]. Nearly 20 compounds were analyzed by GC-MS, and two important compounds were identified by molecular docking. Studies have shown that compounds derived from natural products are more active than compounds of other origins. After years of research and development in drug production, researchers have developed a variety of methods for predicting drug-likeness, such as virtual screening methods, pharmacophore model methods, and machine learning algorithms, which are widely used in various types of compounds [11,12,13,14,15]. The prediction of medicinal properties also has a good effect on the prediction of the drug-like properties of Chinese herbal medicine-derived compounds.

Drug-like prediction plays an important role in improving the efficiency of drug discovery. The consumption of human and material resources can be greatly reduced, which is of great significance for the development of Chinese herbal compound drugs. Based on the medicinal properties of compounds, the research on the pharmacology of traditional Chinese medicine has also made great breakthroughs, and many traditional Chinese Herbal Compound studies are making great progress [16,17]. This article analyzes the advantages and disadvantages of these three methods, and introduces the detailed steps of the virtual screening method. Through the above research, the research prospects in this field are gradually determined.

## 2. Virtual Screening Technology

**Figure 1:**
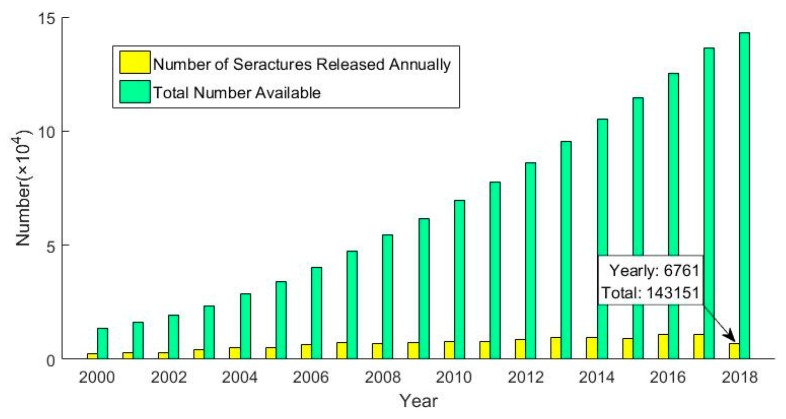
Annual release of compound structure and total structure quantity statistics.

Virtual screening technology is an important part of drug discovery, and this technology brings hope to the goal of significantly reducing time and money spent during drug development. In the current field of drug discovery, virtual screening is often considered to be the best tool for screening large chemical structure databases and candidate drug-specific protein targets [18]. Recently, a variety of prediction methods based on virtual screening have been created. Li Xiaoyi et al. proposed a research idea to reduce adverse drug reactions by lowering the pKa value of the nitrogen atom in the pyrimidine ring [19]. The establishment of molecular data sets follows certain standards, and virtual screening is used to select molecules with the desired pKa values. This method was used to screen out candidate drugs with fewer adverse reactions. Azad L. et al. used a novel heterocyclic derivative as a lead compound to perform virtual screening on 27 compounds previously screened to develop an effective anticancer drug [20]. Compared with other methods, the virtual screening method is more stable and operable for the prediction of Chinese herbal compounds. It distinguishes between active and inactive compounds and is not as dependent on the training set as machine learning methods, while being more accurate. Virtual screening is also a focused and contextual screening method for drug-like compounds [21]. First, we need to be clear that the basic task of drug development is to find an active compound that interacts with and corrects a specific drug target. However, drug targets are usually task-type proteins that perform the physiological activities of cells, and drugs are small molecules that can bind to the characteristic regions of proteins and control their function [22,23]. As shown in Figure 1, the structure of compounds released in recent years is increasing year by year [24]. The rapid development of virtual screening has far exceeded expectations and is gradually enriching the treasure trove of drug data [25,26]. Due to its high throughput, low cost and wide application, the virtual screening technology is consistent with the multi-target and multi-channel characteristics of traditional Chinese medicine compound treatment. Therefore, it is the most basic technology in the prediction of the drug-like properties of traditional Chinese medicine compounds.

Virtual screening involves the following steps: Target selection, ligand selection, molecular docking, virtual screening validation, post-testing and processing. The application examples mentioned below are summarized in Table 1.

### 2.1. Target Selection

Target selection is the first stage in the virtual screening process, and it is the key to whether a drug can be successfully developed. The first step in target selection is the detection of binding sites. Usually, a 3D structure of a protein is known, and its pharmaceutically acceptable property can be evaluated using software [31]. Drug acceptability refers to the ability of this protein to bind drug-like molecules [32,33]. However, it is not usually known whether a ligand can bind to a target protein. In these cases, some calculation tools are needed to calculate and characterize potential binding sites. These algorithms are typically based on geometric features or calculate the interaction energy of the ligand with the acceptor. At present, these two types of algorithms have their own advantages and disadvantages, but based on these two algorithms, 95% of the binding sites can be calculated [34,35]. On the basis of the above, the second step requires preparation of the target. The target standard preparation process mainly includes removing the solvent, removing the ligand molecule, adding a hydrogen atom to establish a bonding sequence, and forming a charge. In general, the preparation of the target greatly affects the enrichment of the final result of the virtual screening.

### 2.2. Ligand Selection

In the selection of ligands, scientific research generally strives for as many varieties of databases as possible. However, in some special cases, it is desirable to limit the amount of the compound to be tested. The choice of ligands often makes it easier for us to manage the breed database. This is often more significant in the study of drug-like prediction of Chinese herbal compounds.

A database of ligands is typically constructed prior to ligand selection. This database can be composed of experimentally known data or drawn from large databases provided by many technology companies. The most commonly used large databases are as follows: (1) NCI, with more than 250,000 compound structures, can be searched for, mainly including the nature and structure of compounds and drug molecules. It is a library of molecular fragments that are commonly used in the industry. (2) ZINC is a free database that provides a search engine and has about 35 million compound structures. (3) MDDR includes information on the structure, biological activity, patent and copyright information, and references for approximately 120,000 drug candidates. These drug candidates are either established or are in clinical research. (4) ADC, the database contains the names of all chemicals currently on the market and provides their structure. (5) TCMCD [36,37], the database is a Chinese medicine chemical database, which mainly includes the structure of Chinese herbal medicine-derived compounds.

In general, there are two ways to reduce the number of ligands in the database. These rejected ligands may not conform to the concept of drugs that scientists have proposed so far, but it is worth noting that the existence of certain good drug candidates that may not comply with these rules cannot be excluded [38,39]. Methods for reducing the number of ligands in a database are generally classified into the simple counting method and the functional group filter method [40,41].

The general counting method mainly considers factors such as partition coefficient, molecular mass, or hydrogen bonding group. Christopher A. Lipinski proposed five rules for drug-like forms: That the number of hydrogen bond donors must be 5 or less, the number of hydrogen bond acceptors must be 10 or less, the molecular weight is less than 500 Da, lipid-water partition coefficients must be 5 or less, and rotation bonds must be 10 or less [27]. After the introduction of the five rules, the theory has undergone many revisions and revisions, and the system is now complete.

Functional group filter methods typically require the use of multiple functional group filters [42]. Functional group filters, as the name suggests, remove the compound that is not suitable for use in a drug. These functional groups often include toxic, mutagenic, teratogenic functional groups; as well as inorganic, insoluble, reactive, and aggregating functional groups.

Virtual screening methods have important guiding significance for early drug discovery, such as the screening of drugs when sample selection and high-quality composite samples are collected. The purpose of this is to exclude some of the impossible medicines, to ensure the quality of the selected compounds, and to ensure the biological activity of the compounds [43].

### 2.3. Molecular Docking

Molecular docking can be performed by selecting the target protein and building up a compound database. Molecular docking requires a lot of expenses and time, so this phase is often essential to virtual screening activities. The aim of molecular docking is to use the preferred orientation of the compound relative to the receptor to predict the binding strength and affinity of the receptor and ligand. Currently, two molecular docking algorithms are widely used to achieve molecular docking, namely search algorithms and scoring functions [44,45]. At the heart of the search algorithm is the calculation of the compounds in different poses to fit the ligand into the binding site of the receptor. The core of the scoring function is to sort and score the different poses and positions generated by the search algorithm for the ligand. This scoring value is idealized to represent the thermodynamic values of the interaction between the acceptor and the ligand. In practical research, the molecular docking score is widely used [46]. Fong P. et al. used molecular docking scores to evaluate the possibility of cordycepin and its derivatives as therapeutic agents for endometrial cancer [28]. In the study of molecular docking evaluation of 31 compounds, the compound numbered MRS5698 was finally successfully screened as a candidate molecule. The molecule has drug molecular characteristics and is highly safe. Ai H. et al. conducted a study on the inhibition of various influenza viruses by Chinese medicine [29]. The study utilizes structure-based molecular docking techniques to screen for more than 10,000 molecular structures from the two databases of the Traditional Chinese Medicine Systems Pharmacology Database and Analysis Platform, from which molecular structures with potential target inhibitors are obtained. The reverse docking technique is then used to verify the tightness of the resulting molecular structure. Finally, 22 molecules capable of stably binding to the target molecule were obtained.

### 2.4. Virtual Screening Validation

Many stages in virtual screening rely on many parameters, so for verification of virtual screening, designing a protocol to verify these parameters should be considered first. In general, the verification of virtual screening generally has the following three aspects, namely the quality of the molecular connection, the accuracy of the fraction, and the ability to distinguish between active and inactive compounds [47].

First, the verification of the quality of the molecular connection requires a re-molecular docking to compare the re-docked structure with the known structure. The standard method of comparison is to calculate the root mean square deviation between the two. The results of the re-docking test are primarily related to the level of the docking model and the quality of the receptor model. It is worth noting, however, that each docking model must use a scoring function to select between various combinations during the search, ultimately choosing the most reasonable solution [48]. Because of this, the re-docking test is also associated with the quality of the scoring function used in conjunction with the search algorithm.

Second, the evaluation of the accuracy of the scoring function is also very important in the virtual screening verification process. For the evaluation of the project, the experimental dissociation constant and the experimental inhibition constant were mainly tested. These constants are directly related to the binding free energy, and the IC_50_ can be calculated by these two values [49]. IC_50_ refers to the semi-inhibitory concentration of the antagonist being measured. It can indicate that a drug or substance (inhibitor) is half the amount that inhibits certain biological processes (or certain substances contained in the procedure, such as enzymes, cellular receptors, or microorganisms) [50,51]. Independent of the scoring unit, higher precision means an increased correlation with the binding affinity of the molecule. Under the guidance of this idea, a linear fit between the score and the experimental value allows prediction of the quality of the virtual screening, since a good fit means that the relative affinities of a series of ligands are well reproduced. The commonly used method is the least squares method, which produces a linear regression equation in which the r free value indicates the quality of the fit. This statistical variable can be compared and selected between different scoring functions, and can be extended to other parameters applied in the virtual filter.

Finally, the distinction between active and inactive compounds in the evaluation of the project is the most important part of the entire validation process. Quantitative virtual screening the ability to evaluate active and inactive molecules is a central element in evaluating virtual screening performance. In the verification, we hope to see that the score of the active molecule should be significantly better than that of the inactive molecule, and the worst score of the active substance should be better than the best score of the inactive substance. However, in fact, there is often a significant overlap between active molecules and inactive molecules, which is often solved by the selection threshold method in practice.

**Figure 2:**
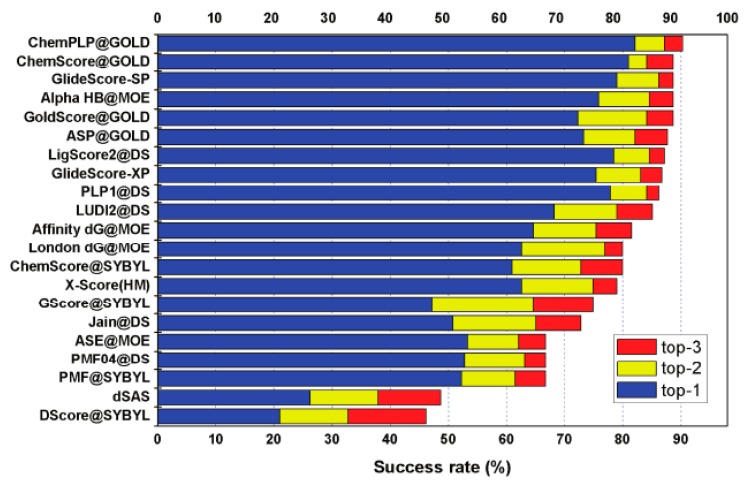
The results diagram of 21 scoring functions in CASF [52].

In the study of scoring function, Chinese researchers have done a lot of excellent work and made outstanding contributions to the development of the scoring function. The research team of the Computational Chemical Biology of the Shanghai Institute of Organic Chemistry of the Chinese Academy of Sciences has long conducted a related study on the scoring function under the leadership of the head of the project. The research group has studied the problem of the size and quality of the scoring function data set and the ambiguity of the standard function of the scoring function. This mainly involves two projects. Project 1 created a database called PDBbind, the first database to link protein-ligand complexes in the Protein Database (PDB) with the experimental data system. The data set provided by PDBbind has been used in many computational and statistical studies on protein-ligand interactions. In particular, it has become the primary data resource for the development of scoring functions. Project 2 established a benchmark for the evaluation of the scoring function (CASF).The main principle is to separate the scoring process from the sampling process so that the scoring function can assess the quality in a more pure environment [52,53,54,55]. The test results of 21 scoring functions in CASF are shown in Figure 2.

### 2.5. Post-Testing and Processing

After the entire virtual screening process, there are usually undesired false positives in the highest score list obtained. In order to overcome these problems, post-filter and co-product scores are often used [56].

The simplest post filter methodology is visual inspection. Visual inspection can accurately determine poor metal coordination and poorly oriented hydrogen bonds [57,58]. However, for large-scale detection, it is obvious that this method is not realistic. In order to solve this problem, we introduced the concept of a polymeric molecule, which is obviously clustering compounds that are similar to each other in the final hit list, and then visually detecting the representative compounds therein [59].

The method of co-product scoring was first proposed by Charifson, who used the docking ligands with their previously set internal docking tools to re-engage the docking ligand conformation with 2–3 scoring functions. Then use the first N% in the shared list. The study finally came to the conclusion that the combined use of scoring functions can significantly improve the elimination rate of false positives. Onawole A.T. et al. used the co-product score method to evaluate the three drug candidates currently selected for the treatment of Ebola virus, and successfully tested that compound SC-2 can be well embedded in Ebola virus trimersugar [30]. On the protein, it may be a good candidate for inhibiting the Ebola virus.

## 3. Introduce Other Prediction Methods of Herbal Compounds in Chinese Medicinal Herbs

### 3.1. Pharmacophore Model

The pharmacophore model: This screening method is mainly aimed at determining whether the molecular structure of the compound exists in the structural fragment common to the drug-like molecule [60,61,62].

In the early studies of the pharmacophore model, scientists focused only on the pharmacophore itself. Bemis et al. found a large amount of information available in the corresponding analysis of the molecular structure of drugs [63]. Their research focused on the analysis and comparison of a large number of drug molecular structures, and concluded that drug molecules have a large degree of structural conservation [64]. The structurally conservative meaning is that certain structures are very similar between drug molecules [65]. Although this method can promote the discovery of lead compounds to some extent, it is difficult to ensure that molecules that are not structurally conserved have no drug-like properties. Similarly, this kind of research cannot form a relatively complete set of identification rules. Wang et al. combined statistically relevant methods to compare a variety of predicted molecules with known drug molecules for more comprehensive attributes [66]. Research suggests that this analogy can preliminarily determine whether a molecule is drug-like. The number of samples in this study is large, and the results have a certain degree of credibility. The study found that the currently marketed drugs and the drug molecules in clinical trials have more than 50% similarity compared to the top50 molecular fragments in the statistical table [67]. By comparing the ratios of different compound molecular fragment types in drug/drug databases and non-pharmaceutical databases, it is possible to determine to some extent which substructure fragments have better drug-like properties.

**Figure 3:**
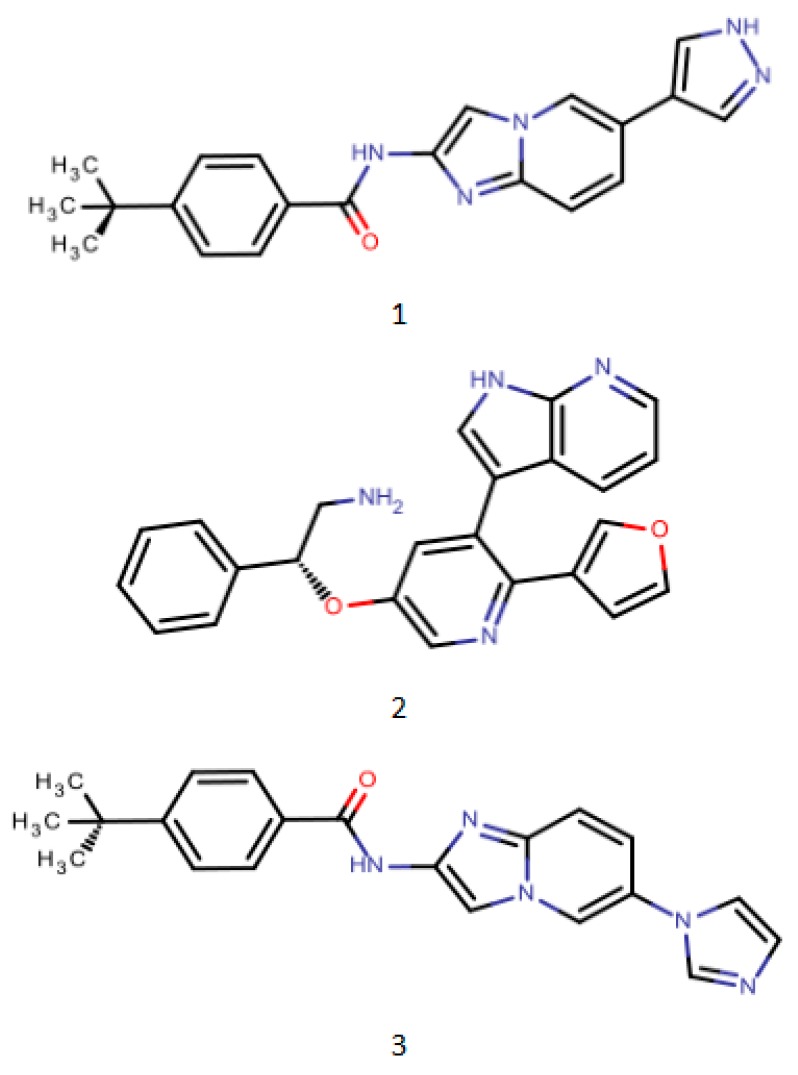
PharmaGistpharmacophore model development training set representative compound structure schematic (1. PDB ID: 4BNH 2. PDB ID: 4BID 3. PDB ID: 3VW6).

In recent years, scientists have paid more attention to the versatility and variability of pharmacophore models [68]. Starosyla et al. studied a three-dimensional pharmacophore model for the development of inhibitors of apoptosis signaling regulator 1 (ASK1), called the PharmaGistprogram [69]. The location of the pharmacophore features in the model corresponds to the conformation of the ASK1 high activity inhibitor, which interacts with the binding site of ASK1. The resulting pharmacophore model accurately predicts both active and inactive compounds and can be used to discover virtual screening of novel ASK1 inhibitors. Several representative composite structures of the PharmaGistpharmacophore model development training set are shown in Figure 3. In order to design a new pharmacophore model with cytotoxicity against K562 cells, Vrontaki et al. generated a 3D pharmacophore model and a three-dimensional structure-activity relationship model study of 33 novel Abl kinase inhibitors [70]. The benzylthiochalcone synthesized by the team is a five-point pharmacophore with a hydrogen bond acceptor, two hydrophobic groups and two aromatic rings as pharmacophores. The pharmacophore model can also be used for the alignment of 33 compounds in the CoMFA/CoMSIA assay. Shang J. et al. used chemical informatics to study the physical and chemical structures and pharmacological pharmacophores of terrestrial and marine natural products, and found that most terrestrial natural products and marine natural products have drug-like characteristics [71]. Marine natural products are more medicinal. Terrestrial and marine natural products have a great potential to become drug guides and even drug candidates.

This method can help define the structure of its drug molecule, which is of great significance for the structural design of the drug, and can also help build a new drug self-contained chemical library. However, this method does not actually distinguish between its drug and non-pharmaceutical properties. We can obtain some important structural information related to drug-like by studying the structural features in drug molecules. However, since there is no versatility standard for selecting molecular, it is difficult to determine whether a structure is a drug-like structure by studying the molecular structure of a drug. In addition, for most molecular fragments, drug molecules and non-drug molecules do not have a clear boundary. This is the main drawback of this method. The application examples mentioned above are summarized in Table 2.

### 3.2. Machine Learning Method

In recent years, AlphaGo based on Deep Learning has consistently defeated the famous Korean Go player Li Shishi and the famous Chinese Go player KeJie [72]. Medical robots developed by Watson and the Massachusetts Institute of Technology team have applied artificial neural networks to disease prediction research [73]. This once again brings machine learning to the public eye. Machine learning algorithms have been widely used in many fields, such as financial analysis, aerospace industry, autonomous driving technology, health care [74], bioinformatics [75], etc. Numerous machine learning methods have also been applied to the study of compound drug prediction, and these methods have shown good results. Machine learning methods use mathematical modelling to find correlations between specific activities or classifications of a group of compounds and their characteristics [76]. There are several methods currently used: Support vector machine (SVM), artificial neural network (ANN), genetic algorithm (GA), recursive algorithm (RP) [77,78,79,80].

The machine learning method for compound drug prediction can be traced back to 1998, when scientists used the Bayesian network method to develop a network model that distinguishes between drug-like and non-drug-like molecules. This model is based on the learning of 3500 drug molecules in the CMC database and 3500 non-drug molecules in the ACD database. The learned network model has accurate predictive performance for more than 90% of the molecules in the source database. Applying this network model to predict the molecules in the MDDR database, the result is that 80% of the molecules have drug-like factors. Since then, many methods have been developed to use neural networks to construct networks for the study of compound drug properties. Ekins et al. developed a Bayesian machine learning model for screening active compounds for the treatment of neural tube defects caused by trypanosomacruzi [81]. The five compounds screened in this study were effective in mouse model experiments, and the best-performing compounds had a cure success rate of 85.2%.

Neural network algorithms have certain advantages in comparison with several other algorithms, but other algorithms have many other advantages. For example, the decision tree and graph model in the recursive partitioning algorithm are more easily understandable. Schneider et al. have established a network model based on recursive partitioning algorithms based on 3117 drugs and 2238 non-pharmaceuticals, but the effect is not particularly ideal [82]. The most widely accepted method at present is the support vector machine method, which is superior to most supervised learning algorithms. It has now been widely adopted for compound drug analysis. The main advantage of the support vector machine method is that the accuracy is improved by a step compared to the neural network algorithm and the recursive segmentation algorithm. Previous studies have used neural networks and support vector machines to construct two network models, and then compare the accuracy of the two. The accuracy of support vector machines is significantly higher than that of neural networks. The relevant experimental data is that the accuracy of the support vector machine algorithm for the training set and the test set is 84.9% and 75.0%, respectively, while the accuracy of the neural network algorithm is 76.6% and 60.8%, respectively. In recent years, research has generally not been limited to a single machine learning method, and multiple methods are often combined to propose a new prediction model. Yosipof et al. used a variety of different machine learning methods to form a new integrated learning method called AL Boost [83]. The AL Boost model combines decision trees, random forest (RF), support vector machine (SVM), artificial neural network (ANN), k nearest neighbor (kNN), and logistic regression models. The actual test shows that the AL Boost prediction model can not only improve the predictive power, but also reduce the bias. Its resolution exceeds 0.81, which has higher sensitivity and specificity than a single model. MaZiRenWan is a Chinese herbal medicine that can effectively relieve functional constipation. Tao Huang et al. analyzed the active compounds in MaZiRenWan and the biological targets they act on using a methodological system called focused network pharmacology [84]. The method incorporates a variety of machine learning methods for predicting possible targets for representative compounds. Experiments have shown that active compounds in MaZiRenWan can enhance colonic peristalsis by acting on multiple targets. Ginkgo biloba has a wide range of applications in the treatment of cardiovascular and cerebrovascular diseases in Chinese medicine, but the mechanism of action of its active ingredients is not clear. Yingfeng Yang et al. used a machine-based C-P network analysis and C-P-T network analysis method to predict and analyze the extracted active compounds [85]. The experiment successfully explained the mechanism of action of Ginkgo biloba leaves for the treatment of cardiovascular and cerebrovascular diseases.

The machine learning-based predictive model is significantly better than the traditional virtual filter in the ability to distinguish between active and inactive compounds. However, the bottleneck of the current machine learning algorithm is how to solve the problem of selecting a training set [86]. Current research has found that the types of molecules in training, including the number and relative balance of drug-like and non-drug-like molecules, have a significant impact on the predictive performance of the algorithm in the future. The application examples mentioned above are summarized in Table 3.

## 4. Conclusions

After thousands of years of development, Chinese medicine is still widely used in clinical treatment, and in some medical fields, it has obvious advantages compared with Western medicine. Chinese medicine is more than just a single clinical science. It has its own uniqueness in prevention science and macro medicine compared with Western medicine. Chinese herbal medicine plays a leading role in the clinical treatment of traditional Chinese medicine. However, due to its diversity and complex composition, its therapeutic mechanism has so far been unclear. One of the solutions to the above problems is to predict the drug-like properties of Chinese herbal medicine-derived compounds. At present, we have entered the era of big data. How to standardize and accurately define the medicinal properties of Chinese herbal medicines has become a obstacle regarding whether Chinese medicine can be scientific and can go global.

At present, the main methods for predicting the medicinal properties of Chinese herbal medicines are virtual screening methods, known drug characterization methods, machine learning algorithms, and blood-brain barrier permeability prediction methods. These methods are most widely used by virtual screening methods because of the low error level of this method and because it has more stability and operability. It distinguishes between active and inactive compounds and is not as dependent on the training set as the machine learning method, and its accuracy is moderate. However, we have seen that this method still has certain limitations, and it is not completely accurate for predicting the drug-like properties of Chinese herbal compounds. Therefore, in future research, we should combine the strengths of several other methods to make the performance of traditional Chinese medicines more stereoscopic under the premise of ensuring accurate testing of the medicinal properties of Chinese herbal medicines. Chinese herbal medicine is the most important part of traditional Chinese medicinal treatment. As traditional Chinese medicine workers, we have the responsibility to explain the treatment mechanism of traditional Chinese medicine more clearly and to make Chinese medicine more scientific. Let Chinese medicine, the treasure of Chinese civilization, be more digital in this era of big data, more evidence-based, and rational, allowing Chinese medicine to contribute more to the world’s medical development and people’s health.

## Figures and Tables

**Table 1:** The application examples of virtual screening technology.

Author	Title	Application	Ref.
Li, X.; Kang, H.; Liu, W.; Singhal, S.; Jiao, N.; Wang, Y.; Zhu, L.; Zhu, R.	In silico design of novel proton-pump inhibitors with reduced adverse effects.	Virtual screening is used to select molecules with the desired pKa values	[19]
Azad, I.; Nasibullah, M.; Khan, T.; Hassan, F.; Akhter, Y.	Exploring the novel heterocyclic derivatives as lead molecules for design and development of potent anticancer agents.	Used a novel heterocyclic derivative as a lead compound to perform virtual screening on 27 compounds previously screened to develop an effective anticancer drug	[20]
McKerrow, J.H.; Lipinski, C.A.	The rule of five should not impede anti-parasitic drug development.	Proposed five rules for drug-like	[27]
Fong, P.; Ao, C.N.; Tou, K.I.; Huang, K.M.; Cheong, C.C.; Meng, L.R	Experimental and in silico analysis of cordycepin and its derivatives as endometrial cancer treatment.	Used molecular docking scores to evaluate the possibility of cordycepin and its derivatives as therapeutic agents for endometrial cancer	[28]
Ai, H.; Wu, X.; Qi, M.; Zhang, L.; Hu, H.; Zhao, Q.; Zhao, J.; Liu, H.	Study on the mechanisms of active compounds in traditional Chinese medicine for the treatment of influenza virus by virtual screening.	The study utilizes structure-based molecular docking techniques to screen for more than 10,000 molecular structures	[29]
Onawole, A.T.; Kolapo, T.U.; Sulaiman, K.O.; Adegoke, R.O.	Structure based virtual screening of the ebola virus trimeric glycoprotein using consensus scoring.	Used the co-product score method to evaluate the three drug candidates currently selected for the treatment of the Ebola virus	[30]

**Table 2:** The application examples of pharmacophore model.

Author	Title	Application	Ref.
Bemis, G.W.; Murcko, M.A.	Properties of known drugs. 2. Side chains.	found a large amount of information available in the corresponding analysis of the molecular structure of drugs	[63]
Wang, J.; Hou, T.	Drug and drug candidate building block analysis.	combined statistically relevant methods to compare a variety of predicted molecules with known drug molecules for more comprehensive attributes	[66]
Starosyla, S.A.; Volynets, G.P.; Bdzhola, V.G.; Golub, A.G.; Protopopov, M.V.; Yarmoluk, S.M.	Ask1 pharmacophore model derived from diverse classes of inhibitors.	The location of the pharmacophore features in the model corresponds to the conformation of the ASK1 high activity inhibitor, which interacts with the binding site of ASK1	[69]
Shang, J.; Hu, B.; Wang, J.; Zhu, F.; Kang, Y.; Li, D.; Sun, H.; Kong, D.X.; Hou, T.	Cheminformatic insight into the differences between terrestrial and marine originated natural products.	used chemical informatics to study the physical and chemical structures and pharmacological pharmacophores of terrestrial and marine natural products	[71]

**Table 3:** The application examples of machine learning method.

Author	Title	Application	Ref.
Ekins, S.; de Siqueira-Neto, J.L.; McCall, L.I.; Sarker, M.; Yadav, M.; Ponder, E.L.; Kallel, E.A.; Kellar, D.; Chen, S.; Arkin, M.	Machine learning models and pathway genome data base for trypanosomacruzi drug discovery.	developed a Bayesian machine learning model for screening active compounds for the treatment of neural tube defects caused by trypanosomacruzi	[81]
Schneider, B.; Balbas-Martinez, V.; Jergens, A.E.; Troconiz, I.F.; Allenspach, K.; Mochel, J.P.	Model-based reverse translation between veterinary and human medicine: The one health initiative.	have established a network model based on recursive partitioning algorithms based on 3117 drugs and 2238 non-pharmaceuticals, but the effect is not particularly ideal	[82]
Yosipof, A.; Guedes, R.C.; Garcia-Sosa, A.T.	Data mining and machine learning models for predicting drug likeness and their disease or organ category.	used a variety of different machine learning methods to form a new integrated learning method called AL Boost	[83]
Huang, T.; Ning, Z.; Hu, D.; Zhang, M.; Zhao, L.; Lin, C.; Zhong, L.L.D.; Yang, Z.; Xu, H.; Bian, Z.	Uncovering the mechanisms of Chinese herbal medicine (mazirenwan) for functional constipation by focused network pharmacology approach.	The method incorporates a variety of machine learning methods for predicting possible targets for representative compounds	[84]
Zhou, W.; Wang, J.; Wu, Z.; Huang, C.; Lu, A.; Wang, Y.	Systems pharmacology exploration of botanic drug pairs reveals the mechanism for treating different diseases.	used a machine-based C-P network analysis and C-P-T network analysis method to predict and analyze the extracted active compounds	[85]
